# Comparison of surgical and oncological outcomes between different surgical approaches for overweight or obese cervical cancer patients

**DOI:** 10.1007/s11701-024-01863-4

**Published:** 2024-03-04

**Authors:** Wanli Chen, Rong Wang, Jialin Wu, Yingyu Wu, Lin Xiao

**Affiliations:** Department of Gynecology, The First Affiliated Hospital of Chongqing Medical University, Chongqing, 400016 China

**Keywords:** Cervical cancer, Radical total hysterectomy, Obesity, Outcomes, Complications

## Abstract

The purpose was to investigate the safety and advantages of different surgical approaches applied to overweight or obese cervical cancer patients by comparing their surgical and oncological outcomes. This is a retrospective cohort study. 382 patients with a body mass index of at least 24.0 kg/m^2^ and stage IB-IIA (The International Federation of Gynecology and Obstetrics, FIGO 2009) cervical cancer were enrolled, and then were divided into three groups: open radical hysterectomy (ORH) group, laparoscopic radical hysterectomy (LRH) group, and robot-assisted radical hysterectomy (RRH) group according to the surgical approach. IBM SPSS version 25.0 was used to analyze data. There were 51 patients in ORH group, 225 patients in LRH group and 106 patients in RRH group. In the comparison of surgical outcomes, compared to LRH and ORH, RRH had the shortest operating time, the least estimated blood loss, the shortest postoperative hospital stay, and the shortest recovery time for bowel function (*P* < 0.05). In the comparison of postoperative complications, ORH has the highest rate of postoperative infection and wound complication compared to LRH and RRH (*P* < 0.05), and RRH has the highest proportion of urinary retention. After a median follow-up time of 61 months, there was no statistically significant difference between the three groups in terms of 5-year overall survival (OS) rate and 5-year recurrence-free survival (RFS) rate, (*P* = 0.262, *P* = 0.453). In patients with overweight or obese cervical cancer, the long-term outcomes of the three surgical approaches were comparable, with RRH showing significant advantages over ORH and LRH in terms of surgical outcomes.

**Supplementary Information:**

The online version contains supplementary material available at 10.1007/s11701-024-01863-4.

## Introduction

The International Cancer Centre reports that an estimated 604,000 women worldwide will be diagnosed with cervical cancer and approximately 342,000 women will die from the disease in 2020 [1]. Currently, according to the NCCN and FIGO guidelines, surgery plays an important role in the treatment strategy for stage IB-IIA cervical cancer. ORH, LRH, and RRH are widely used in the treatment of cervical cancer. In 2018, LACC trial showed that ORH had higher disease-free survival, higher overall survival (OS) than minimally invasive surgery [2]; however, this conclusion is currently controversial. There are no uniform findings on the surgical outcomes and surgical complications associated with the three surgical approaches to cervical cancer. Previous study found that being overweight or obese was a risk factor for adverse events within 6 months postoperatively, but no further analysis was done [3].

Some studies have found that “obesity” plays an important role in the development, treatment and prognosis of cervical cancer [4, 5]. During surgery, overweight and obese patients have more comorbidities, difficulties in managing intraoperative anesthesia, difficulties in exposing the surgical field, high risk of postoperative complications and difficulties in recovery [6, 7].

There is limited information and data about three surgical procedures for overweight or obese patients with cervical cancer [8]. In this study, we compare the surgical outcomes and oncological outcomes between the three surgical procedures, so as to provide an objective basis for preoperative decision-making in obese or overweight cervical cancer patients.

## Materials and methods

### Patients and data collection

The study protocol was approved by the Human Ethics Committee of The First affiliated Hospital of Chongqing Medical University. Data were retrospectively collected from 2014.01.01 to 2019.12.31 on patients who underwent surgery at the Department of Gynecology, the First Hospital of Chongqing Medical University. Information was obtained from hospital inpatient records, outpatient records, and telephone follow-up, which ended in May 2023. Inclusion criteria were: (1) overweight or obese patients with cervical cancer. According to the characteristics of the Asian population, a body mass index (BMI) ≥ 24.0 kg/m^2^ is called overweight and a BMI ≥ 28.0 kg/m^2^ is called obese [9]; (2) FIGO 2009 stage IB-IIA; (3) completion of radical total hysterectomy + pelvic/para-aortic lymph node dissection; (4) postoperative pathological type of squamous, adenocarcinoma or adenosquamous carcinoma. Exclusion criteria were: (1) those with a uterus larger than 3 months' gestation; (2) those who had undergone other procedures unrelated to the treatment of cervical cancer; (3) those who have combined other malignancies; (4) those who had undergone preoperative radiotherapy; (5) those with severe pelvic and abdominal adhesions. Postoperative treatment was decided by this medical team from our gynecology department. Patients are divided into an ORH group, LRH group, and RRH group according to the surgical approach.

### Variables

(1) Baseline characteristics: age, BMI, history of pelvic and abdominal surgery, comorbidities, postoperative staging, pathology report; (2) surgical outcomes: 1) perioperative outcomes: lumpectomy to open, operating time, estimated blood loss, transfusion, time to return of bowel movement, postoperative hospital stay, no. of lymph nodes retrieved, 2) complications: intraoperative pelvic and abdominal organ injury, surgery-related nerve injury, urinary retention, thrombosis, infection, urinary fistula, pelvic lymphocele; (3) oncological outcomes: OS: time from surgery to death for any cause or latest follow-up; RFS: RFS defined as the time from surgery to recurrence. Operating time: time from skin incision to closure; estimated blood loss (EBL): total suction volume − intraoperative flush volume + gauze containing blood. Time to return of bowel movement: time to first postoperative evacuation. Urinary retention is when the patient cannot urinate smoothly, bladder residual urine volume was ≥ 100 ml as determined by B ultrasound, or cannot self-discharge and need to reset the catheter or need for intermittent clean catheterization [10]. Infection: clinical diagnosis in the medical record during hospitalization or with pathogenic evidence. Pelvic lymphocele: cystic mass in the pelvis detected by ultrasound or CT or MRI. Surgery-related nerve injury: pain or weakness in the groin area, or lower limbs during the postoperative hospital stay, and to exclude pain or weakness due to thrombosis or pre-existing neurological pathology. Wound complication: including poor healing of the incision such as postoperative fat liquefaction and infection.

### Statistics

Continuous variables were described using median (interquartile spacing) and mean ± standard deviation. Data normality using the Kolmogorov–Smirnov test were not normally distributed, and Kruskal–Wallis test was used to compare differences between groups. Categorical variables were described using frequency (frequencies) and differences of qualitative results were analyzed by Chi-square test or Fisher exact test. RFS and OS were estimated using the Kaplan–Meier method. Univariable analyses were performed to compare the three study groups, and the Cox proportional hazards model was used. All prognostic variables showing significance in the univariable analyses were then included in a multivariable analysis involving the Cox proportional hazards model. *P* values of 0.05 in the two-sided tests were considered significant. All statistical analyses were performed using SPSS 25.0 for Windows.

## Results

### Table 1 shows baseline characteristics in the study

**Table 1:** Baseline characteristics in the study (*n* = 382)

Characteristics	ORH (*n* = 51)	LRH (*n* = 225)	RRH (*n* = 106)	*H*/*X*^2^ value	*P* value
Age (y)	47.0 (43.0–52.0)	48.0 (43.0–53.0)	50.0 (45.0–55.0)	3.223	0.200
	48.1 ± 8.2	48.3 ± 9.0	49.4 ± 9.2		
BMI (kg/m^2^)	25.6 (24.5–26.7)	25.7 (24.8–27.3)	26.0 (24.8–27.4)	2.652	0.266
	25.8 ± 1.5	26.3 ± 2.2	26.5 ± 2.3		
Previous abdominal surgery				1.105	0.593
No	35 (68.6%)	155 (68.9%)	67 (63.2%)		
Yes	16 (31.4%)	70 (31.1%)	39 (36.8%)		
Medical disease				0.971	0.606
No	39 (76.5%)	172 (76.4%)	86 (81.1%)		
Yes	12 (23.5%)	53 (23.6%)	20 (18.9%)		
Neoadjuvant chemotherapy				0.800	0.683
No	25 (49.0%)	112 (49.8%)	58 (54.7%)		
Yes	26 (51.0%)	113 (50.2%)	48 (45.3%)		
FIGO stage (2009)				1.468	0.479
IB1 or IIA1	34 (66.7%)	136 (60.4%)	60 (56.6%)		
IB2 or IIA2	17 (33.3%)	89 (39.6%)	46 (43.4%)		
Lymph node involvement				1.325	0.570
Negative	48 (94.1%)	200 (88.9%)	94 (88.7%)		
Positive	3 (5.9%)	25 (11.1%)	12 (11.3%)		
Stromal invasion				6.143	0.189
≤1/3	28 (54.9%)	107 (47.6%)	46 (43.4%)		
1/3 ~ 2/3	16 (31.4%)	73 (32.4%)	29 (27.4%)		
≥2/3	7 (13.7%)	45 (20.0%)	31 (29.2%)		
Tumor histology				8.283	0.016
Squamous	38 (74.5%)	185 (82.2%)	97 (91.5%)		
Others^a^	13 (25.5%)	40 (17.8%)	9 (8.5%)		
Lymphovascular space invasion				8.610	0.014
Negative	49 (96.1%)	196 (87.1%)	84 (79.2%)		
Positive	2 (3.9%)	29 (12.9%)	22 (20.8%)		

^a^Adenocarcinoma or adenosquamous carcinoma

A total of 382 overweight or obese stage IB-IIA cervical cancer patients were enrolled in this study, 51 (13.4%) in the ORH group, 225 (58.9%) in the LRH group and 106 (27.7%) in the RRH group. The median age of all patients was 49 years (28–76 years) and the median BMI was 25.8 kg/m^2^ (24.0–34.0 kg/m^2^). Table 1 shows the characteristics of the study cohort. There were no statistically significant differences between three groups in any of the following (*P* > 0.05): demographic factors (including age, BMI, history of previous abdominal surgery, preoperative neoadjuvant chemotherapy, bulky tumor (greater than 4 cm), and comorbidities between the three groups). None of the patients had a postoperative pathology report indicating positive margins or parametrial infiltration. Squamous carcinoma is the predominant pathological type in all three groups. The proportion of patients with adenocarcinoma or adenosquamous carcinoma was lower in the RRH group than in the ORH and LRH groups, and the difference was statistically significant (*P* = 0.006, *P* = 0.026). The rate of lymphovascular space invasion was higher in the RRH group than in the ORH group, and the difference was statistically significant (*P* = 0.008).

### Table 2 shows surgical outcomes in the study

**Table 2:** Surgical outcomes in the study cohort (*N* = 382)

	ORH (*n* = 51)	LRH (*n* = 225)	RRH (*n* = 106)	*H*/*X*^2^ value	*P* value
Operating time (min)	230.0 (205.0–260.0)	215.0 (180.0–257.0)	182.0 (155.0–210.0)	47.069	<0.001
	231.9 ± 37.4	222.7 ± 56.9	184.3 ± 38.5		
Estimated blood loss (mL)	400.0 (200.0–600.0)	100.0 (50.0–200.0)	80.0 (50.0–100.0)	104.699	<0.001
	462.9 ± 274.1	148.4 ± 121.0	93.7 ± 78.6		
Transfusion	7 (13.7%)	6 (2.7%)	6 (5.7%)	10.904	0.004
Return of bowel movement (d)	2.0 (2.0–3.0)	2.0 (2.0–2.0)	2.0 (2.0–2.0)	32.495	<0.001
	2.45 ± 0.7	1.92 ± 0.6	1.90 ± 0.6		
Postoperative hospital stay (d)	11.0 (9.0–14.0)	9.0 (8.0–13.0)	8.0 (7.0–10.0)	23.012	<0.001
	11.3 ± 3.4	10.2 ± 3.1	9.3 ± 4.4		
No. of lymph nodes retrieved	35 (29–43)	34 (27–44)	37 (28–44)	1.147	0.564
	37 ± 13	36 ± 13	37 ± 13		
Surgery-related nerve injury	2 (3.9%)	16 (7.1%)	6 (5.7%)	0.815	0.656
Injury to abdominal organ	0 (0.0%)	0 (0.0%)	3 (2.8%)	5.701	0.038
Urinary retention	7 (13.7%)	43 (19.1%)	35 (33.0%)	10.529	.005
Thrombosis	0 (0.0%)	6 (2.7%)	0 (0.0%)	3.005	0.172
Wound complication	16 (31.4%)	1 (0.4%)	0 (0.0%)	58.950	<0.001
Fistula	0 (0.0%)	3 (1.3%)	0 (0.0%)	1.155	0.710
Infection	25 (49.0%)	48 (21.3%)	20 (18.9%)	19.693	<0.001
Pelvic lymphocele	17 (33.3%)	48 (21.3%)	43 (40.6%)	13.886	0.001

None of the LRH and RRH groups required conversion to laparotomy. The number of lymph nodes retrieved was similar between the three surgical approaches (*P* = 0.564). Operating time, EBL, blood transfusion during hospitalization, time to return of bowel movement, and postoperative hospital stay were statistically significant difference among the three groups (*P* < 0.05), and a two-by-two comparison between the three groups is shown in supplementary file. (1) The operating time is significantly shorter in the RRH group than in the ORH and LRH groups (182.0 min VS 230.0 min, *P* < 0.001 and 180.0 min VS 215.0 min, *P* < 0.001); (2) the EBL was least in the RRH group compared to ORH group and LRH group (80.0 ml VS 400.0 ml, *P* < 0.001 and 80.0 ml VS 100.0 ml, *P* < 0.001); (3) the rate of transfusion during hospitalization was lower in the LRH group than in the ORH group (*P* = 0.004); (4) the postoperative hospital days was shorter in the RRH group than in the ORH and LRH groups (8.0 days VS 11 days, *P* < 0.001 and 8.0 days VS 9 days, *P* < 0.001); (5) the ORH group took longer to return of bowel movement than the LRH and RRH groups (2.45 days VS 1.92 days, *P* < 0.001 and 2.45 days VS 1.90 days, *P* < 0.001).

There was no significant difference between the 3 groups for surgery-related nerve injury, fistula, and thrombosis (*P* = 0.656, *P* = 0.172, *P* = 0.710). Three patients in the LRH group developed ureterovaginal fistulas within 1 month postoperatively with vaginal fluid, which were confirmed by the urological CTU. Intraoperative pelvic and abdominal organ injury, urinary retention, infection, and pelvic lymphocele were significantly different among the three groups (*P* < 0.05), and a two-by-two comparison between the three groups is shown in supplementary file. (1) All intraoperative pelvic and abdominal organ injury patients presented in the RRH group, healed finally; (2) the rate of urinary retention was significantly higher in the RRH group than in the ORH and LRH groups (33.0% VS 19.1%, *P* = 0.006 and 33.0% VS 13.7%, *P* = 0.012); (3) the rate of wound complication was significantly higher in the ORH group than in the LRH and RRH groups (31.4% VS 0.4%, *P* < 0.001 and 31.4% VS 0.0%, *P* < 0.001). Only one patient in LRH group had poor vaginal incision healing, while the rest occurred in the abdominal incision; (4) the rate of infection was significantly higher in the ORH group than LRH and RRH (49.0% VS 21.3%, *P* < 0.001 and 49.0% VS 8.9%, *P* < 0.001); (5) the rate of pelvic lymphocele was significantly lower in the LRH group than in the RRH group (*P* < 0.001).

### Oncological outcomes

**Fig. 1:**
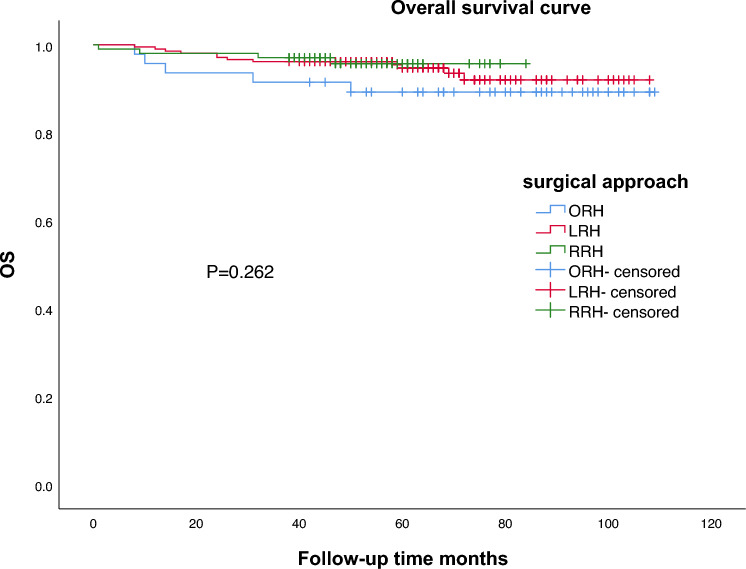
Survival curves based on surgical approach (*n* = 359). *OS* overall survival

**Fig. 2:**
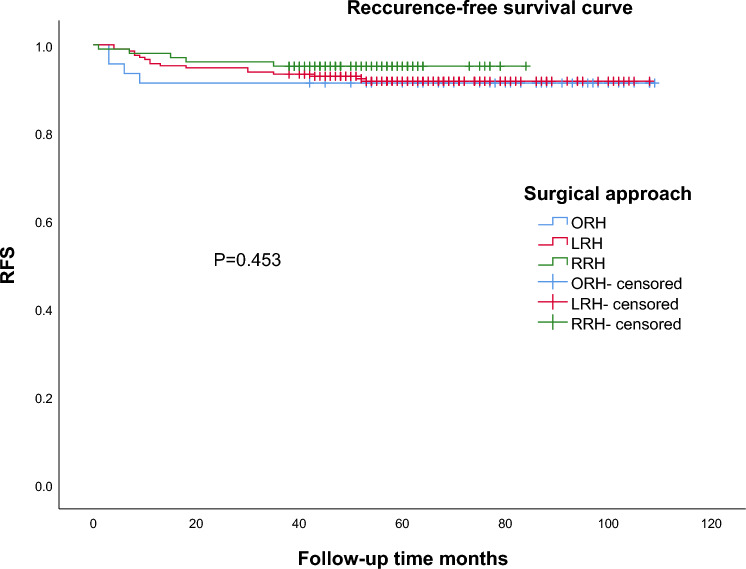
Survival curves based on surgical approach (*n* = 359). *RFS* recurrence-free survival

The median follow-up time was 61.0 months (50.0–73.8 months). During the follow-up period, a total of 23 patients (6.0%) were lost, 21 patients died and 26 patients recurrence. The 5-year OS was 89.3% for ORH group, 94.7% for LRH group and 95.7% for RRH group, with no significant difference among three groups (*P* = 0.262) (Fig. 1); and the 5-year RFS was 89.3% for ORH group, 91.7% for LRH group and 95.1% for RRH group, with no significant difference among three groups (*P* = 0.453) (Fig. 2). In the univariable analysis, more EBL, longer postoperative hospital days, wound complication, stromal invasion (≥2/3), lymph node involvement, and lymphovascular space invasion were significantly associated with poorer OS (*P* < 0.05); in the multivariable analysis, more EBL, longer postoperative hospital, stromal invasion (>1/3), lymph node involvement, and lymphovascular space invasion were significantly associated with poorer OS (*P* < 0.05) in Table 3. In the univariable analysis, more EBL, longer postoperative hospital days, stromal invasion (≥2/3), and lymph node involvement were significantly associated with poorer RFS (*P* < 0.05). In the multivariable analysis, more EBL, longer postoperative hospital, stromal invasion (>1/3), lymph node involvement, and lymphovascular space invasion were significantly associated with poorer RFS (*P* < 0.05), in Table 4.

**Table 3:** Factors associated with overall survival in the study cohort

Variable	Univariable analysis		Multivariable analysis	
	HR (95% CI)	*P* value	HR (95% CI)	*P* value
Surgical approach (ORH)				
Surgical approach (LRH)	0.682 (0.115–4.040)	0.673	–	–
Surgical approach (RRH)	2.286 (0.288–18.154)	0.434	–	–
Operating time	1.003 (0.993–1.013)	0.544	–	–
Estimated blood loss	1.005 (1.001–1.008)	0.006	1.004 (1.002–1.005)	<0.001
Postoperative hospital stay	1.363 (1.140–1.629)	0.001	1.086 (1.017–1.159)	0.014
No. of lymph nodes retrieved	0.972 (0.924–1.022)	0.268	–	–
Return of bowel movement	2.213 (0.705–3.069)	0.051	1.597 (0.777–3.281)	0.203
Surgery-related nerve injury	1.895 (0.416–8.640)	0.409	–	–
Injury to abdominal organ	0.000 (0.000)	0.966	–	–
Urinary retention	0.714 (0.215–2.32)	0.583	–	–
Thrombosis	1.590 (0.162–15.595)	0.690	–	–
Transfusion	0.235 (0.015–3.662)	0.301	–	–
Wound complication	0.014 (0.001–0.311)	0.007	0.230 (0.027–1.985)	0.181
Fistula	0.000 (0.000)	0.992	–	–
Infection	1.569 (0.515–4.782)	0.428	–	–
Pelvic lymphocele	0.941 (0.313–2.830)	0.914	–	–
Stromal invasion (≤ 1/3)			–	–
Stromal invasion (1/3 ~ 2/3)	3.773 (0.868–16.390)	0.076	4.658 (1.245–17.426)	0.022
Stromal invasion (≥ 2/3)	7.330 (1.476–36.410)	0.015	6.034 (1.444–25.221)	0.014
Lymph node involvement	6.017 (1.947–18.598)	0.002	5.420 (1.959–14.997)	0.001
Lymphovascular space invasive	4.304 (1.166–15.885)	0.028	2.995 (1.072–8.362)	0.036
Tumor histology (squamous)				
Tumor histology (others^a^)	2.170 (0.697–6.758)	0.181	–	–

^a^Adenocarcinoma or adenosquamous carcinoma

**Table 4:** Factors associated with recurrence-free survival in the study cohort

Variable	Univariable analysis		Multivariable analysis	
	HR (95% CI)	*P* value	HR (95% CI)	*P* value
Surgical approach (ORH)				
Surgical approach (LRH)	1.376 (0.265–7.152)	0.704	–	–
Surgical approach (RRH)	1.626 (0.240–11.000)	0.618	–	–
Operating time	1.002 (0.994–1.011)	0.584	–	–
Estimated blood loss	1.003 (1.000–1.006)	0.028	1.003 (1.001–1.004)	<0.001
Postoperative hospital stay	1.188 (1.034–1.364)	0.015	1.066 (0.998–1.139)	0.057
No. of lymph nodes retrieved	0.968 (0.931–1.007)	0.104	–	–
Return of bowel movement	1.714 (0.870–3.378)	0.120	–	–
Surgery-related nerve injury	1.453 (0.373–5.666)	0.590	–	–
Injury to abdominal organ	0.000 (0.000)	0.971	–	–
Urinary retention	1.190 (0.446–3.172)	0.729	–	–
Thrombosis	1.073 (0.125–9.196)	0.949	–	–
Transfusion	0.710 (0.079–6.412)	0.760	–	–
Wound complication	0.118 (0.009–1.607)	0.109	–	–
Fistula	0.000 (0.000)	0.991	–	–
Infection	1.008 (0.385–2.637)	0.987	–	–
Pelvic lymphocele	0.639 (0.239–1.712)	0.373	–	–
Stromal invasion (≤ 1/3)				
Stromal invasion (1/3 ~ 2/3)	2.805 (0.939–8.377)	0.065	3.883 (1.358–11.101)	0.011
Stromal invasion (≥ 2/3)	3.588 (1.032–12.476)	0.044	4.477 (1.398–14.33)	0.012
Lymph node involvement	3.595 (1.350–9.569)	0.010	3.222 (1.337–7.762)	0.009
Lymphovascular space invasion	2.685 (0.972–7.418)	0.057	2.454 (1.002–6.006)	0.049
Tumor histology (squamous)				
Tumor histology (others^a^)	1.956 (0.758–5.043)	0.165	–	–

^a^Adenocarcinoma or adenosquamous carcinoma

## Discussion

Previous studies have shown that EBL and postoperative hospital stay in robotic assisted surgery are significantly better or not worse than in open and conventional laparoscopic surgery [7, 11–13]. In this study, target population is overweight or obese cervical cancer patients, we found that EBL, bowel function recovery time, postoperative hospital days, wound complications, and infections in RRH were significantly lower than ORH; EBL, bowel function recovery time, postoperative hospital days in RRH were significantly lower than LRH, RRH has significant benefits in patient perioperative outcomes which were similar to previous studies. In this study, it was shown that the operative time of RRH was significantly shorter than that of ORH and LRH, which is not similar to previous studies [13–15], the studies included in the META analysis of Kampers and Guo are older, mostly before 2000–2010, the earliest year of research in this study is 2014, RRH has increased significantly in volume in the last few years, and proficiency overcomes many of the difficulties of operations. Similar to previous studies [16, 17], RRH compares to LRH, the surgical field of view is controlled by the surgeon and the surgeon’s hand–eye coordination is more coordinated, has a 3D visual effect, which makes the intraoperative dissection of blood vessels and nerves more delicate and accurate [18, 19], and the machine arm’s ability to thick abdominal wall support and exposure of the narrow abdominal cavity with fat accumulation overcomes the disadvantages of LRH, it may account for the shorter operative time as well as less bleeding with RRH compared to LRH. Less bleeding and shorter operating time are associated with fewer postoperative complications [20, 21], so overweight or obese cervical cancer patients undergoing robot-assisted radical hysterectomy had the lowest incidence of postoperative infections, faster recovery, fewer postoperative hospital days and reduced use of medical resources, such as blood transfusions and antibiotics. Overweight or obese cervical cancer patients with long incision faced healing difficulties in open surgery, such as fat liquefaction and incisional infection [7, 22, 23]. In this study, only 0.3% in the LRH group had poor healing of the incision where the drainage tube was located, compared to 31.4% in ORH group, suggesting that minimally invasive surgery in overweight or obese cervical cancer patients can almost avoid poor healing of the incision after surgery.

This study found that the proportion of urinary retention was significantly higher in the RRH group than in the ORH. Bladder dysfunction is a common complication after radical total hysterectomy + pelvic lymph node dissection because of intraoperative damage to the pelvic autonomic nerves that innervate the bladder muscles, urethral sphincter and pelvic floor fascia [24]. Ralph et al. concluded that patients are more likely to experience impaired bladder sensation and increased residual urine after radical total hysterectomy compared to total hysterectomy [25], and Ercoli et al. concluded that the degree of postoperative bladder denervation depends on the amount of vaginal, paravaginal and parametrial tissue removed [26]. Therefore, it may suggest more adequate scope for robotic assisted surgical excision of parametrial tissue, vagina in cervical cancer patients with overweight or obese. The results of this study are consistent with the literature, which reports a 1–58% incidence of pelvic lymphocele after radical cervical cancer surgery [27]. The number of lymph nodes removed was an independent risk factor for the formation of pelvic lymphocele, and the more the number of lymph nodes removed, the higher the probability of postoperative pelvic lymphocele [28]. In this study, the incidence of pelvic lymphocele was found to be higher in the RRH group than in the other two groups. Although there was no significant difference in the number of lymph nodes removed between the three groups in this study, the median number of lymph nodes removed in the RRH group was higher than in the other two groups, so we can be assumed that in the fatty pelvis, pelvic lymph nodes were more thoroughly removed by robotic surgery. The incidence of postoperative urinary retention as well as pelvic lymphocele was higher in the RRH group than in the other two groups, both suggest that it may be due to more adequate surgical coverage and have a positive impact on long-term survival in surgically difficult overweight or obese cervical cancer.

Robotic assisted surgery shows more advantages in overweight or obese cervical cancer patients. But ureterovaginal and vesicovaginal fistulas are complications associated with the extensive separation of the ureter and peri-vesical tissue during radical hysterectomy. In previous study, it was found that the risk of urinary complications after conventional laparoscopic surgery was greater than open surgery [29], with no difference between conventional and robotic laparoscopic surgery [30]. All the patients included in this study had postoperative ureterovaginal fistulas in LRH group, but the number of cases was low and the difference between the groups was not statistically significant.

LACC [2] and SUCCOR [31] trials mutually validate superior oncological survival outcomes of ORH over minimally invasive surgery in early-stage cervical cancer. Laparoscopic and robotic techniques have not been prospectively studied and have only been used as an emerging technique in radical hysterectomy in cervical cancer, possibly ignoring the principle of tumor-free operation, e.g., destruction of tumor tissue during the use of transcervical uterine manipulators and tumor dissemination caused by CO_2_ pneumoperitoneum has been ignored [32]. Only overweight or obese patients with stage IBI–IIA2 cervical cancer were included in this study, and the results showed that the 5-year OS and RFS in RRH and LRH were comparable to ORH. More than 50 percent of the cases in this study underwent preoperative neoadjuvant chemotherapy, which may reduce tumor activity, reduce the likelihood of intraoperative dissemination, and increase the feasibility of complete intraoperative resection, so that the margins of the cuts in all cases in this study were negative; After hysterectomy, strict disinfection of the vaginal stump is performed using iodophor, the lesion specimen is wrapped in a cup of transcervical uterine manipulators and removed, and postoperative pelvic lavage is performed using copious amounts of saline, which reduce the chance of tumor implantation; postoperative supplemental treatment plan based on sedlis principles in conjunction with pathology report, preoperative imaging, physical examination and intraoperative specimen dissection; our center is highly skilled in performing RRH and has extensive experience in the treatment of cervical cancer, which ensures adequate resection coverage and appropriate complementary treatment. These may be the reason why the survival outcome of minimally invasive surgery in our center is similar to that of ORH.

Previous studies suggest that obesity is associated with longer operative times, more blood loss and a higher risk of complications [7, 33]. For overweight or obese cervical cancer patients, the perioperative advantages of minimally invasive surgery are greater and the RRH advantage is more prominent and can reduce the physical burden of the surgeon. The worse survival outcomes of minimally invasive surgery than open surgery may be due to neglect of the tumor-free principle, etc. Many scholars are also calling for the use of minimally invasive surgery in cervical cancer not to be rejected outright, and that more research should be done to explore the reasons for the differences between the two as well as to find solutions. In overweight or obese patients, robotic surgery offers surprising benefits to patients, and a prospective trial of robotics versus open radical cervical cancer surgery is currently underway and deserves attention [34].

This is a retrospective study, which may have resulted in some bias because patients were not randomly assigned at the time of surgery, but may have been selected based on certain patient characteristics. However, the larger sample size in this study allows for less bias. WHO defines overweight as BMI ≥ 25.0 kg/m^2^ and obesity as BMI ≥ 30.0 kg/m^2^; however, studies had shown that among Chinese with the same level of BMI, Chinese have higher levels of body fat and are more likely to have a combination of obesity-related diabetes and cardiovascular disease [35]; therefore, the inclusion criteria for this study were set according to the Chinese expert consensus on overweight as BMI ≥ 24.0 kg/m^2^ and obesity as BMI ≥ 28.0 kg/m^2^.

## Conclusion

In summary, robotic assisted surgery has the least EBL, the shortest operative time, the least rate of postoperative infection, and the shortest postoperative days for more difficult procedures such as overweight or obese cervical cancer. There was no significant relationship between the surgical approach and postoperative death or recurrence in overweight or obese cervical cancer patients, and OS or RFS at 5 years in RRH and LRH was comparable to that of ORH.

## Supplementary Information

Below is the link to the electronic supplementary material.

Supplementary file1 (DOCX 13 kb)

## Data Availability

No datasets were generated or analyzed during the current study.
