# Supplementary material for: Comparison of surgical and oncological outcomes between different surgical approaches for overweight or obese cervical cancer patients
Source: J Robot Surg. 2024 Mar 4;18(1):107. doi: 10.1007/s11701-024-01863-4 (PMC10912340; doi:10.1007/s11701-024-01863-4)
Supplement: Supplementary file 1 — Supplementary file1 (DOCX 13 kb) [file 11701_2024_1863_MOESM1_ESM.docx]

Table 4. Comparison among groups

|  | LRH-RRH | |  | ORH-LRH | |  | ORH-RRH | |
| --- | --- | --- | --- | --- | --- | --- | --- | --- |
|  | H/X2 | P value |  | H/X2 | P value |  | H/X2 | P value |
| Operating time | 75.525 | <.001 |  | 36.448 | .100 |  | 111.973 | <.001 |
| Estimated blood loss | 55.372 | <.001 |  | 135.228 | <.001 |  | 190.601 | <.001 |
| Transfusion | - | .210 |  | - | .004 |  | - | .120 |
| Return of bowel movement | 2.750 | <.001 |  | 77.721 | <.001 |  | 80.471 | <.001 |
| Postoperative hospital stay | 46.712 | <.001 |  | 36.850 | 0.090 |  | 83.562 | <.001 |
| Injury to abdominal organ | - | .032 |  | * | * |  | - | .551 |
| Urinary retention | 7.738 | .006 |  | 0.813 | 0.427 |  | 6.541 | .012 |
| Wound complication | - | 1.000 |  | - | <.001 |  | 37.029 | <.001 |
| Infection | 0.268 | .663 |  | 16.382 | <.001 |  | 15.310 | <.001 |
| Pelvic lymphocele | 13.370 | <.001 |  | 3.325 | .098 |  | 0.763 | .483 |

- : Represents Fisher's exact test. *: Represents no intraoperative pelvic or abdominal organ injury between ORH and LRH groups and cannot be compared between groups.
